# A novel homozygous frameshift mutation likely causing nonsense-mediated mRNA decay in an Algerian kindred with CD19 complex deficiency

**DOI:** 10.3389/fimmu.2025.1634146

**Published:** 2025-09-05

**Authors:** Brahim Belaid, Koon-Wing Chan, Lydia Lamara Mahammed, Daniel Leung, Sara Makhloufi, Fadila Bendaoud, Hassiba Sakhri, Lilya Meriem Berkani, Ines Allam, Fatma Merah, Hadda Baaziz, Bernice Lo, Jaime Sou Rosa Duque, Yu Lung Lau, Reda Djidjik

**Affiliations:** ^1^ Department of medical immunology, Beni Messous University Hospital Center, Algiers, Algeria; ^2^ Faculty of Pharmacy, The University of Health Sciences, Algiers, Algeria; ^3^ Department of Pediatrics and Adolescent Medicine, School of Clinical Medicine, Li Ka Shing Faculty of Medicine, The University of Hong Kong, Hong Kong, Hong Kong SAR, China; ^4^ Department of pediatrics, Batna Women’s & Children’s Hospital, Batna, Algeria; ^5^ Faculty of Medicine, The University of Batna, Batna, Algeria; ^6^ Department of Pediatrics, Batna University Hospital Center, Batna, Algeria; ^7^ Research Branch, Sidra Medicine, Doha, Qatar; ^8^ College of Health and Life Sciences, Hamad Bin Khalifa University, Doha, Qatar

**Keywords:** primary antibody deficiency, CD19, CD21, CD81, B lymphocyte

## Abstract

**Background:**

CD19 is an essential component of a membrane protein complex on B cells, which also includes complement receptor 2 (CD21), CD81, and CD225. It amplifies B cell receptor (BCR) signaling by recruiting regulatory molecules and facilitating the phosphorylation of key kinases. Mutations in the *CD19* gene disrupt the integrity of this complex and impair BCR signaling, ultimately leading to antibody deficiency.

**Purpose:**

we report here a novel mutation in the *CD19* gene in two patients from consanguineous Algerian kindred.

**Methods:**

We conducted a comprehensive analysis of the clinical, genetic, and immunological characteristics of two siblings with CD19 deficiency.

**Results:**

Both siblings began experiencing upper and lower respiratory tract infections in early childhood. Over time, the older sibling developed recurrent fungal and viral skin infections, as well as episodes of pyelonephritis. Whole exome sequencing identified a novel homozygous mutation in the *CD19* gene, leading to an out-of-frame translation predicted to trigger nonsense-mediated decay and result in absent gene expression. Flow cytometry revealed a complete absence of CD19 and reduced CD21 expression on CD20^+^ B cells in both siblings, while CD81 expression remained normal. Despite normal total peripheral B cell counts, the older patient exhibited reduced memory B cells. Additionally, both patients displayed circulating autoantibodies and an increased frequency of circulating follicular helper T cells.

**Conclusion:**

These findings highlight the critical role of CD19 not only in the initial activation of B lymphocytes by T-dependent antigens, but also in the maturation and/or selection of activated B cells within the memory compartment.

## Introduction

**Figure 1:**
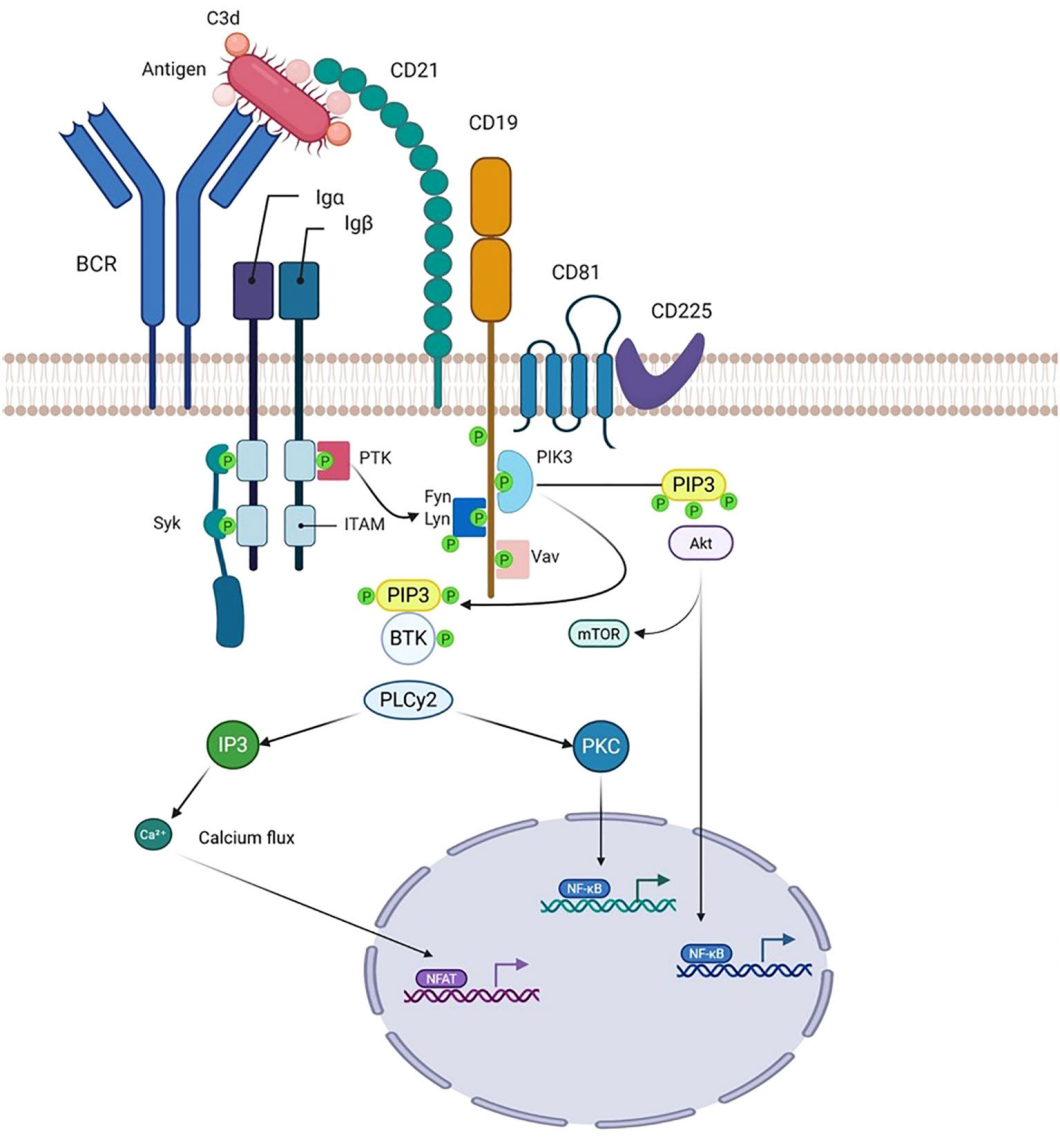
Structure of the B cell receptor (BCR)–CD19 complex and proposed mechanisms by which the CD21/CD19 co-receptor complex amplifies BCR signaling. Co-ligation of the B cell receptor (BCR) and the CD21–CD19 complex by a C3d–antigen complex enables a Src-family tyrosine kinase associated with CD79 to phosphorylate specific tyrosine residues within the cytoplasmic domain of CD19. Phosphorylated CD19 then serves as a scaffold to recruit key SH2 domain–containing signaling molecules to the BCR complex, thereby amplifying and propagating the initial BCR-mediated signal through multiple intracellular signaling cascades. Created in BioRender. djidjik, r. (2025) https://BioRender.com/m0dksll.

CD19 is a B cell surface antigen and glycoprotein that is broadly expressed throughout B cell development—from early progenitor stages to terminal differentiation into plasma cells (1). It is encoded by the *CD19* gene, located on the short arm of chromosome 16 (16p11.2) (1, 2). CD19 functions as a core component of a multimolecular membrane complex, along with CD21, CD81, and CD225, which is expressed on mature B lymphocytes (**Figure 1**). Signaling through this complex is essential for B cell development, differentiation, and maturation (1, 3). Structurally, CD19 is a 95-kDa type I transmembrane glycoprotein belonging to the immunoglobulin superfamily. It contains two extracellular Ig C2-like domains and a large cytoplasmic tail of approximately 240 amino acids, which harbors at least nine tyrosine residues—several of which are crucial for mediating downstream signaling events (1, 4–6).

The CD19 complex acts as a crucial co-stimulatory element during antigen recognition via the B cell receptor (BCR). It also bridges innate and adaptive immunity by enhancing B cell activation and promoting adaptive immune responses through complement-dependent opsonization (7). Co-ligation of CD21 and the BCR has been shown to significantly enhance BCR signaling. Although CD21 possesses only a short cytoplasmic tail, it is the long intracellular domain of CD19 that serves as the primary transducer of downstream signals, ultimately promoting B cell activation and proliferation. Consequently, antigens opsonized with complement fragment C3d—which engage both CD21 and the BCR—can amplify signaling cascades by transmitting activation signals through CD19 (8).

Over the past three decades, a limited number of individuals have been reported with genetic defects in the *CD19* gene, resulting in impaired B cell differentiation and function, and leading to an autosomal recessive form of hypogammaglobulinemia that closely resembles common variable immunodeficiency (CVID) (9, 10). In addition, mutations in the *CD21* and *CD81* genes—due to their essential roles in the assembly and function of the CD19 complex—can also lead to defective antibody production, primarily as a result of inadequate antigen responses and impaired immunological memory (11–15). Patients with deficiencies in CD19, CD21, or CD81 share similar clinical and immunological phenotypes, though disease severity can vary. Symptoms typically begin in childhood and most commonly include recurrent upper and lower respiratory tract infections, sometimes accompanied by skin and gastrointestinal infections (9, 11–14, 16–18). Despite these immune impairments, patients generally have normal numbers of circulating B lymphocytes, marked by CD20 expression, but exhibit little or no CD19 on the B cell surface, along with reduced frequencies of transitional and memory B cells (18).

Here, we report two siblings harboring a homozygous nucleotide deletion in exon 2 of the *CD19* gene. Premature termination codons may trigger nonsense-mediated mRNA decay (NMD), a surveillance pathway that degrades aberrant transcripts to prevent production of truncated proteins (19–21). This mechanism likely underlies the lack of CD19 expression observed in our patients. In contrast to previously described cases of CD19 deficiency, neither patient exhibited overt hypogammaglobulinemia, except for a moderate reduction in IgG levels in one sibling who continued to experience recurrent skin infections despite immunoglobulin replacement therapy.

## Materials and methods

### Clinical evaluation

Clinical and demographic information, including age, sex, and familial relationships, was collected from the two siblings with CD19 deficiency and their parents.

### Blood samples and ethical approval

Peripheral blood samples were obtained from the CD19-deficient siblings, their unaffected siblings and parents, as well as age-matched healthy controls, following informed consent and in accordance with the guidelines approved by the Ethics Committee of the University Medical Center of Beni Messous.

### Serum immunoglobulin determination and autoantibody assays:

Serum immunoglobulin levels (IgG, IgA, IgM) were quantified using nephelometry (BN ProSpec^®^ analyzer; Siemens Healthcare Diagnostics, Marburg, Germany), while serum IgE levels were measured by chemiluminescent immunoassay (CLIA) using the Immulite^®^ 2000 XPi system (Siemens Healthcare Diagnostics, Germany).

Sera (serum samples) were screened for circulating autoantibodies associated with both organ-specific and non-organ-specific autoimmune diseases. Detection was performed qualitatively by indirect immunofluorescence (IIF) and quantitatively by enzyme immunoassays (EIA), including enzyme-linked immunosorbent assay (ELISA) and chemiluminescent immunoassay (CLIA). For connective tissue disease screening, sera were tested for anti-nuclear antibodies (ANAs) using IIF at a 1:80 dilution on HEp-2 cells (INOVA Diagnostics, San Diego, CA, USA) and for IgM rheumatoid factor. Positive ANA patterns were further titrated by serial dilution and analyzed for specific autoantibodies against Ro/SSA (52 and 60 kDa), La/SSB, Sm, Sm/RNP, Scl-70, dsDNA, and Jo-1 using EIA (Quanta-Lyzer 160/240; CA, USA).

For ulcerative colitis and vasculitis, sera were screened for antineutrophil cytoplasmic antibodies (ANCA) by IIF at a 1:20 dilution on human neutrophil slides (INOVA Diagnostics). Coeliac disease screening involved testing IgA anti-tissue transglutaminase (tTG) antibodies on the Alegria analyzer (Orgentec Diagnostika, Mainz, Germany). Autoantibodies associated with autoimmune hepatitis and primary biliary cirrhosis—including F-actin, liver-kidney microsomal antigen (LKM), soluble liver antigen (SLA), antimitochondrial antibodies (AMA), GP210, and SP100—were measured by ELISA (INOVA Diagnostics, San Diego, CA, USA). For autoimmune thyroiditis, thyroid peroxidase antibodies (TPOAb) and thyroglobulin antibodies (TgAb) were assessed using the Immulite 2000 XPi system (Siemens Healthcare Diagnostics, Germany).

### Flow cytometric immunophenotyping

To characterize the major lymphocyte subsets (T, B, and NK cells), and to analyze the T and B cell compartments along with CD19, CD21, and CD81 expression on B cells, the following monoclonal antibodies (mAbs) were used: CD3 (SK7), CD4 (SK3), CD8 (SK1), CD19 (SJ25C1), CD20 (L27), CD21 (B-ly4), CD24 (ML5), CD25 (M-A251), CD31 (WM59), CD38 (HB7), CD45 (HLe-1), CD45RA (L48), CD56 (NCAM16.2), CD81 (JS-81), CD127 (HIL-7R-M21), CCR7 (IA6-2), IgD (IA6-2), TCRαβ (IP26), and FOXP3 (C259D/C7) (all from BD Biosciences); CD16 (3G8), CD45RO (UCHL1), and CD27 (M-T271) (Beckman Coulter); and CXCR5 (51505) (Bio-Techne, R&D Systems Inc.). Detailed antibody panels are provided in **Supplementary Table S1**.

For surface marker analysis, 100 µL of whole blood was incubated with monoclonal antibodies for 20 minutes at room temperature in the dark. Red blood cells were lysed, followed by washing. For intracellular staining, cells were fixed, permeabilized, washed, and incubated with the relevant mAbs for 30 minutes before final washing and resuspension in phosphate-buffered saline (PBS). Samples were acquired on a BD FACS Lyric™ flow cytometer (Becton Dickinson, San Jose, CA, USA) and analyzed using BD FACSuite™ Software (v2.1) and FlowJo v10.8 (BD Biosciences).

Gating strategies for T cell subsets included: CD4+ naive T cells (CD4+ CD45RA+ CCR7+), central memory (CD4+ CD45RA– CCR7+), effector memory (CD4+ CD45RA– CCR7–), and terminally differentiated effector memory (TEMRA, CD4+ CD45RA+ CCR7–); CD8+ naive (CD8+ CD45RA+ CCR7+), central memory (CD8+ CD45RA– CCR7+), effector memory (CD8+ CD45RA– CCR7–), and TEMRA (CD8+ CD45RA+ CCR7–) T cells. Regulatory T cells (Tregs) were defined as CD4+ CD25+ CD127 low/– FOXP3+, and T follicular helper (Tfh) cells as CD4+ CD45RO+ CXCR5+.

B cell subsets were classified as follows: naive mature B cells (CD20+ CD27– IgD+), non-class-switched memory B cells (CD20+ CD27+ IgD+), class-switched memory B cells (CD20+ CD27+ IgD–), transitional B cells (CD20+ CD24 hi CD38 hi), plasmablasts (CD20+ CD24– CD38 hi), and autoreactive CD21 low CD38 low B cells.

The surface expression of CD19, CD21, and CD81 was assessed in patients, compared to isotype controls, a healthy unrelated control (13-year-old girl), heterozygous carrier parents, and two non-carrier siblings. Treg and Tfh cell frequencies in the two patients and their parents were also compared to five age- and sex-diverse unrelated healthy controls (three females aged 9, 11, and 33 years; two males aged 9 months and 42 years).

### Activation and intracellular cytokine staining

Functionally polarized CD4^+^ T cell subsets—including type 1 helper T (Th1), Th2, and Th17 effector and/or memory cells—are defined by their distinct cytokine secretion profiles. To detect intracellular cytokines, peripheral blood mononuclear cells (PBMCs) were first isolated using a density gradient centrifugation method. PBMCs were stimulated with phorbol 12-myristate 13-acetate (PMA; 50 ng/mL, Sigma-Aldrich) and ionomycin (1 μg/mL, Sigma-Aldrich) in the presence of BD GolgiStop™ (BD Biosciences), and incubated for 5 hours at 37°C with 5% CO_2_.

Following stimulation, cells were fixed using BD Cytofix™ Fixation Buffer containing formaldehyde (BD Biosciences) for 15 minutes at room temperature, then washed twice with FACS buffer (containing bovine serum albumin and sodium azide). Permeabilization was performed using BD Perm/Wash™ Buffer (BD Biosciences) for 20 minutes, followed by two additional washes.

Cells were then stained with fluorochrome-conjugated monoclonal antibodies against intracellular cytokines and surface markers, including anti-IFN-γ (clone B27), anti-IL-4 (MP4-25D2), anti-IL-17A (N49-653), CD3 (SK7), and CD4 (SK3) (all from BD Biosciences). Data acquisition was performed using a BD FACS Lyric™ Flow Cytometer and analyzed with BD FACSuite™ Software v1.2 and FlowJo v10.8 (BD Biosciences).

### Antigen-specific antibody responses

Antibody levels specific to tetanus toxoid, diphtheria toxoid, and pneumococcal capsular polysaccharide (PCP) were quantified using commercial ELISA kits, in accordance with the manufacturers’ instructions. The following kits were used: VaccZyme™ Tetanus Toxoid IgG, VaccZyme™ Diphtheria Toxoid IgG, and VaccZyme™ PCP IgG (all from The Binding Site Group Ltd, Birmingham, UK).

### *In vitro* T cell responses

Peripheral blood mononuclear cells (PBMCs) were isolated from heparinized blood samples of patient 1 and an unrelated healthy control (13-year-old female) using Ficoll-Hypaque density gradient centrifugation. A cell suspension of 1 × 10_6_ PBMCs/mL in phosphate-buffered saline (PBS) containing 0.1% fetal bovine serum (FBS; Invitrogen, Carlsbad, CA, USA) was labeled with 10 µM carboxyfluorescein succinimidyl ester (CFSE; Invitrogen) for 10 minutes at 37°C in the dark. Following incubation, excess dye was removed by washing.

A total of 3 × 10_5_ CFSE-labeled PBMCs were seeded into each well of a 12-well flat-bottom culture plate containing RPMI 1640 medium supplemented with 10% FBS, 100 U/mL penicillin, and 100 µg/mL streptomycin. Cells were stimulated either with 1 µg/mL phytohemagglutinin-M (PHA-M; Sigma-Aldrich) or with anti-CD3/anti-CD28–coated Dynabeads (DB; Gibco). Unstimulated CFSE-labeled PBMCs were cultured under identical conditions as controls.

After incubation, cells were harvested and stained with 7-AAD, anti-CD3, and anti-CD4 antibodies for 30 minutes at room temperature. Following washing, data acquisition was performed using a BD FACSLyric™ flow cytometer (Becton Dickinson, San Jose, CA, USA), and data were analyzed with FlowJo software version 10.8 (BD Biosciences).

### DNA isolation and genetic analysis

Genomic DNA (gDNA) was extracted from whole blood using a previously described protocol (22). Whole-exome sequencing (WES) was conducted on gDNA from the first affected sibling (Patient 1). Identified mutations in the *CD19* gene were subsequently confirmed by Sanger sequencing in both affected siblings. Segregation analysis was performed in the parents and unaffected siblings through targeted amplification and sequencing of exon 2 of the CD19 gene.

## Results

### Clinical features

**Figure 2:**
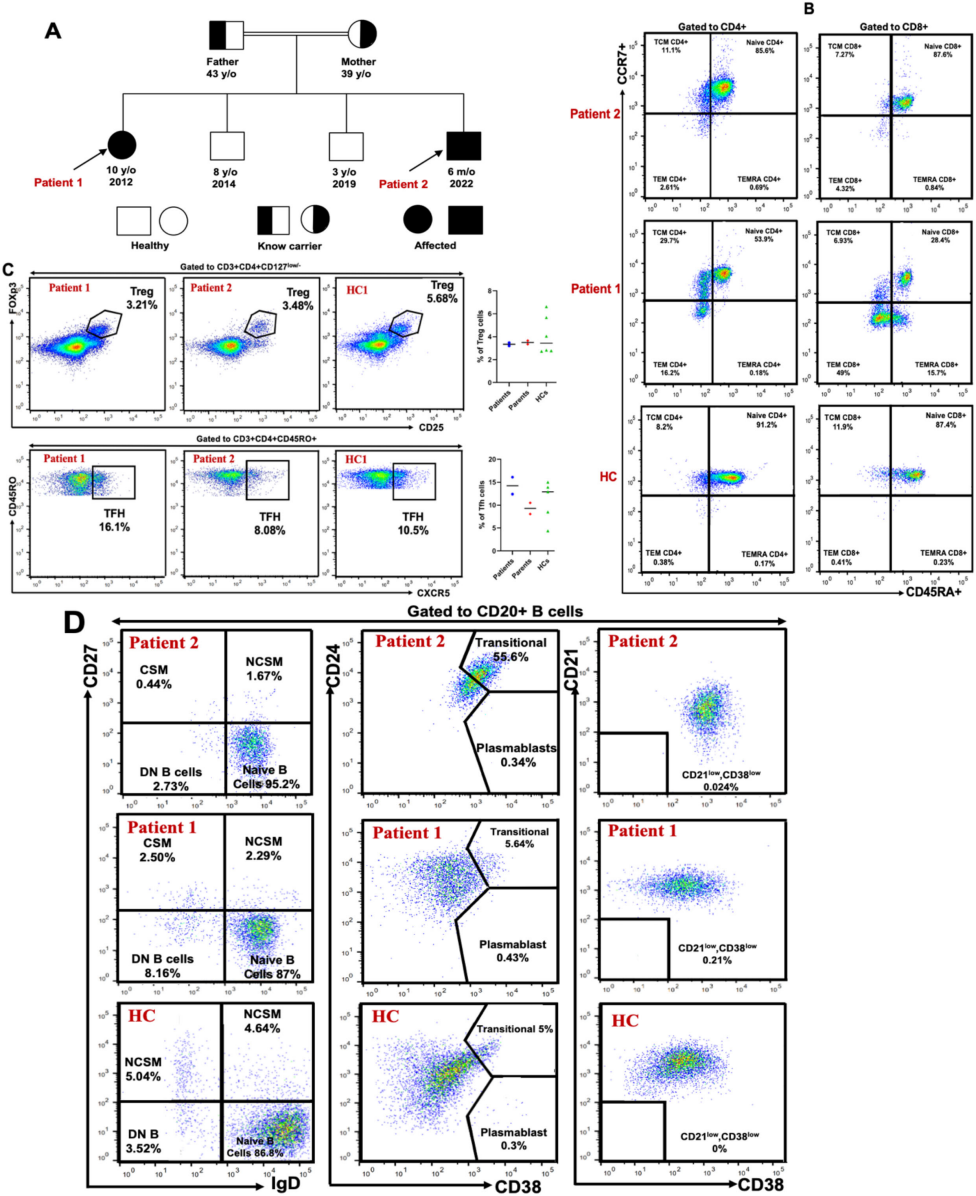
Genetic and immunophenotypic characterization of CD19 deficiency in a consanguineous family. **(A)** Family pedigree. The pedigree illustrates a consanguineous union between the parents, both heterozygous carriers of the c.232delC (p.Leu78Trpfs*52) variant in the *CD19* gene. Two unaffected brothers did not carry the mutation, while both patients were homozygous for the deletion. **(B)** T-cell subpopulations. Flow cytometric analysis of peripheral blood from both patients (1 and 2) and a healthy control showing the distribution of naïve T cells, central memory (TCM), effector memory (TEM), and terminally differentiated effector memory (TEMRA) T cells, based on CD4, CD8, CD45RA, and CCR7 expression within the CD3^+^ T-cell compartment. T-cell subsets were compared to established reference ranges from our laboratory. **(C)** Regulatory and follicular helper T cells. Representative flow cytometry plots showing the frequencies of FOXP3^+^CD25^+^ regulatory T (Treg) cells and CXCR5^+^CD45RO^+^ follicular helper T (Tfh) cells within the CD4^+^ T-cell population. **(D)** B-cell subpopulations. Distribution of transitional, naïve, memory, autoreactive B cells, and plasmablasts in peripheral blood of patients 1 and 2, and a healthy control assessed within the CD20^+^ B-cell compartment using CD21, CD24, CD27, CD38, and IgD markers. B-cell subsets were also compared to established reference ranges from our laboratory.

Both siblings were born to healthy consanguineous Algerian parents (**Figure 2A**). Patient 1 is a 10-year-old girl. Her prenatal and perinatal histories were unremarkable. However, beginning at 8 months of age, she experienced recurrent urinary tract infections and episodes of pyelonephritis, necessitating multiple hospitalizations. No congenital anomalies of the kidneys or urinary tract were identified. From the age of 3 years, she developed recurrent episodes of acute purulent otitis media and sinopulmonary infections, which were managed with antibiotics and did not require hospitalization.

At the age of 6, she presented with an episode of extensive herpetic skin lesions that resolved spontaneously. She also experienced several episodes of mucocutaneous fungal infections, primarily affecting the scalp, skin, and genital area, which responded well to topical antifungal treatment. She received all routine childhood immunizations in accordance with the Algerian national vaccination schedule.

On physical examination, the patient exhibited growth and developmental delays, with a length at -2 standard deviations (SD) and weight at -3 SD. The remainder of the physical examination was unremarkable, including assessments of the lymph nodes, skin, head, heart, neck, oral cavity, and the digestive, urogenital, and respiratory systems.

Patient 2 is a 5-month-old boy who was hospitalized at 5 months of age due to severe pneumonia with pleural effusion and respiratory distress. He was born at term but had a low birth weight (2.1 kg). During the first five months of life, he experienced multiple episodes of upper respiratory tract infections.

On admission, physical examination revealed a high-grade fever (39°C), oxygen saturation of 72% on room air, polypnea at 76 breaths per minute, a hacking cough, and tachycardia with a pulse rate of 140 beats per minute. Growth parameters were significantly below age norms, with a weight of -4 SD and length of -3 SD. Auscultation revealed crackles at the bases of both lungs. Additionally, the patient presented with watery diarrhea. There was no clinical evidence of lymphadenopathy or hepatosplenomegaly.

### Laboratory investigations

For Patient 1, initial laboratory evaluation revealed a hemoglobin level of 13.6 g/dL and a white blood cell (WBC) count of 14,200/mm³, with a differential of granulocytes 47.1%, lymphocytes 46.2%, and monocytes 6.7%. Initial immunological investigations (**Table 1**) showed a moderate decrease in serum IgG at 436 mg/L (reference range: 700–1160 mg/L), while IgA was within normal limits at 144 mg/L (ref: 79–169 mg/L), as were IgM at 42 mg/L (ref: 40–90 mg/L) and IgE at 11.9 IU/mL (ref: <52 IU/mL). Protective antibody titers were markedly reduced, with tetanus and diphtheria toxoid-specific IgG levels being low to undetectable (0.024 IU/mL and 0.001 IU/mL, respectively). Following immunization with Pneumovax^®^23 (Pneumococcal Vaccine Polyvalent), specific IgG antibodies against pneumococcal capsular polysaccharides remained low at 30.8 mg/L. IgG subclass analysis showed a selective deficiency of IgG1.

**Table 1:** Immunological characteristics of two siblings with CD19 deficiency.

Parameter	Patient 1		Patient 2		
	Result	Normal range	Result		Normal range
Immunoglobulin levels					
**IgG (mg/dl)**	**436** 595*	700–1160 690–1150		**1580**	320–520
**IgG1 (mg/dl)**	**284**	432 –1020		N/D	/
**IgG2 (mg/dl)**	89	72 – 430		N/D	/
**IgG3 (mg/dl)**	60	12.7 – 85.3		N/D	/
**IgG4 (mg/dl)**	3	1.9 – 93.2		N/D	/
**IgA (mg/dl)**	144 129*	79–169 68–194		**86.4**	10-46
**IgM (mg/dl)**	42 406*	40–90 390–790		45.4	20-66
**IgE (IU/ml)**	11,9	<52		N/D	/
Lymphocyte subsets					
**CD3^+^ T cells/mm^3^**	**4447**	1200 – 2600		**1482**	2500 – 5600
**CD4^+^ T cells/mm^3^**	**2086**	650 – 1500		**859**	1800 – 4000
**CD4_Naive_CD45RA^+^CCR7^+^ T cells/CD4^+^ T cells%**	53.90	15.5 – 59.4		**85.6**	25.6 – 78.4
**CD4_CM_CD45RA^-^CCR7^+^ T cells/CD4^+^ T cells%**	**29.70**	12.2 – 26.2		11.10	8.3 – 21
**CD4_EM_CD45RA^-^CCR7^-^ T cells/CD4^+^ T cells%**	16.2	10.6 – 34.2		**2.61**	3.4 – 12.4
**CD4_TEMRA_CD45RA^+^CCR7^-^ T cells/CD4^+^ T cells%**	**0.18**	4.5 – 43.6		**0.69**	5.1 – 55.4
**CD4_RTE_CD45RA^+^CD31^+^/CD4^+^ T cells%**	41.52	25.8 – 68		65.57	19.4 – 60.9
**Treg (CD25^++^CD127^-^FOXP3^+^)/CD4^+^ T cells%**	3.21	2.02– 3.6		3.48	2.02– 3.6
**Tfh (CD4^+^ CD45RO^+^ CXCR5^+^)/CD4+T cells%**	**16,1**	8.1 – 14.7		**8,08**	8.1 – 14.7
**CD8^+^ T cells/mm^3^**	**1925**	370 – 1100		537	590 – 1600
**CD8_Naive_CD45RA^+^CCR7^+^ T cells/CD8^+^ T cells%**	28.4	5.5 – 39.7		87.6	8.3 – 64.9
**CD8_CM_CD45RA^-^CCR7^+^ T cells/CD8^+^ T cells%**	6.93	1.2 – 3.8		7.27	1.9 – 9.5
**CD8_EM_CD45RA^-^CCR7^-^ T cells/CD8^+^ T cells%**	49	20.1 – 44.7		**4.32**	5.1 – 41.5
**CD8_TEMRA_CD45RA^+^CCR7^-^ T cells/CD8^+^ T cells%**	15.70	21.5 – 61		0.84	14 – 65.4
**CD4^+^/CD8^+^ Ratio**	1.08	1.5 – 2.9		1.60	1.5 – 2.9
**CD19^+^B cells/mm^3^**	**0**	270 – 860		**0**	430 – 3000
**CD20^+^B cells/mm^3^**	562	270 – 860		**266**	430 – 3000
**B_naive_ (CD27^-^IgD^+^)/CD20^+^ B cells %**	87	44.95 – 75.80		**95.2**	65.54 – 86.62
**B_switched memory_ (CD27^+^IgD^-^)/CD20^+^ B cells %**	**2.5**	5.2 – 12.1		0.44	0.1 – 1.9
**B_unswitched >memory_ (CD27^+^IgD^+^)/CD20^+^ B cells %**	**2.29**	7.5 – 12.4		1.67	1.6 – 4.1
**B_transitional_ (CD24^++^CD38^++^)/CD20^+^ B cells %**	5.64	4.5 – 9.2		**55.6**	8.3 – 15.8
**B_plasmablast_ (CD24^-^CD38^++^)/CD20^+^ B cells %**	**0.43**	0.7 – 3.5		0.34	0.2 – 1.0
**B_CD21(-/low)_ (CD21^low^CD38^low^)/CD20^+^ B cells %**	**0.21**	0.9 – 3.5		**0.024**	0.3 – 4.0
**CD3^-^CD16^+^CD56^+^ NK cells/mm^3^**	115	100 – 480		**119**	170 – 830
Post-vaccination antibody titres					
**Anti-tetanus toxoid IgG (UI/mL)**	**0,027**	≥ 0,1		N/D	/
**Anti-diphtheria toxoid IgG (UI/mL)**	**0,001**	≥ 0,1		N/D	/
Autoantibodies					
**IgG Antineutrophil Cytoplasmic Antibodies (ANCA)**	Negative	<1/20		Negative	<1/20
**IgG anti-nuclear autoantibodies (ANA) on HEp-2**	1/160 (Speckled)	<1/80		Negative	<1/80
**Extractible nuclear antigen (ENA) Screen (U/mL)**	11	<20		10	<20
**IgG anti-dsDNA (IU/mL)**	<10	<30		<10	<30
**IgM Rheumatoid factor (U/mL)**	<10	<20		<10	<20
**IgG anti-F-Actin (U/mL)**	**23**	<20		**39**	<20
**IgG antimitochondrial M2 (U/mL)**	10	<20		17	<20
**IgG anti-SP100 (U/mL)**	7	<20		16	<20
**IgG anti-GP210 (U/mL)**	6	<20		11	<20
**IgG anti- LKM1(U/mL)**	3	<20		8	<20
**IgG anti- SLA (IU/mL)**	7	<20		**60**	<20
**IgG anti-intrinsic factor (U/mL)**	4	<25		6	<25
**IgG anti-parietal cells (U/mL)**	5	<20		5	<20
**IgA anti-transglutaminase (IU/mL)**	6	<10		5	<10
**IgG-anti-thyroid peroxidase (TPO) (IU/mL)**	12	<35		15	<35
**IgG anti-thyroglobulin (TG) (IU/mL)**	10	<40		18	<40

CM, Central memory; EM, effector memory; TEMRA: Terminally differentiated T cells; RTE, Recent Thymic Emigrant, N/D, not done.

Values in bold are abnormal.

*Second data point collected at age 10

Lymphocyte immunophenotyping by flow cytometry demonstrated an absence of CD19+ B cells (0% of total lymphocytes), while CD20+ B cells were present at 11% (absolute count: 562/mm³). T cell populations included CD3+ T cells at 86.8% (4447/mm³), CD4+ T cells at 40.7% (2086/mm³), CD8+ T cells at 37.6% (1925/mm³), with a CD4/CD8 ratio of 1.08. Natural killer (CD56+) cells accounted for 2.2% (115/mm³). Based on these findings, the patient was started on intravenous immunoglobulin (IVIG) therapy at a dose of 0.4 g/kg every 21 days.

Further in-depth immunophenotyping of T and B cell subsets was performed (**Figures 2B–D**). No significant abnormalities were observed in the frequencies of naïve or effector T cell subsets. Regulatory T cells (Tregs) were within normal limits, while T follicular helper (Tfh) cells were slightly elevated compared to age- and sex-matched healthy controls. In the B cell compartment, despite normal total B cell counts, the frequencies of both switched and non-switched memory B cells and plasmablasts were reduced, with a concomitant increase in naïve B cells. These findings suggest an underlying defect in B cell activation and maturation. The near-absent post-immunization antibody responses against tetanus and diphtheria toxoids, as well as persistently low anti-pneumococcal antibody levels, further support this immunological dysfunction.

For Patient 2, laboratory findings revealed a white blood cell (WBC) count of 15,800/mm³ with a differential of 64% neutrophils, 19.9% lymphocytes, 9% monocytes, and 5.4% eosinophils. Hemoglobin was 11.5 g/dL and platelet count were elevated at 788,000/mm³. Serum immunoglobulin levels showed increased IgG and IgA, while IgM was within normal limits. Renal and hepatic function parameters, as well as serum electrolyte levels, were within normal ranges. C-reactive protein (CRP) was markedly elevated at 24 mg/dL (reference: <6 mg/dL). A chest X-ray revealed right-sided pleuropneumonia.

Lymphocyte subset analysis showed normal percentages but decreased absolute counts for all subsets when compared to age-matched reference values: CD3+ T cells at 79.4% (1482/mm³), CD4+ T cells at 46% (859/mm³), CD8+ T cells at 28.7% (537/mm³), CD20+ B cells at 14.2% (266/mm³), and CD16+CD56+ NK cells at 6.4% (119/mm³). Notably, B cells lacked surface expression of CD19. Post-vaccination antibody responses could not be evaluated, as the patient had only received the BCG vaccine.

Further immunophenotyping of T and B cell compartments revealed an expansion of transitional B cells and a reduction in plasmablasts, while other lymphocyte subpopulations were within normal limits (**Figures 2B–D**). Based on these immunologic findings and the positive family history, a diagnosis of CD19 deficiency was established. The patient was initiated on immunoglobulin replacement therapy at a dose of 0.4 g/kg every three weeks.

### Genetic and molecular characterization of the CD19 defect

**Figure 3:**
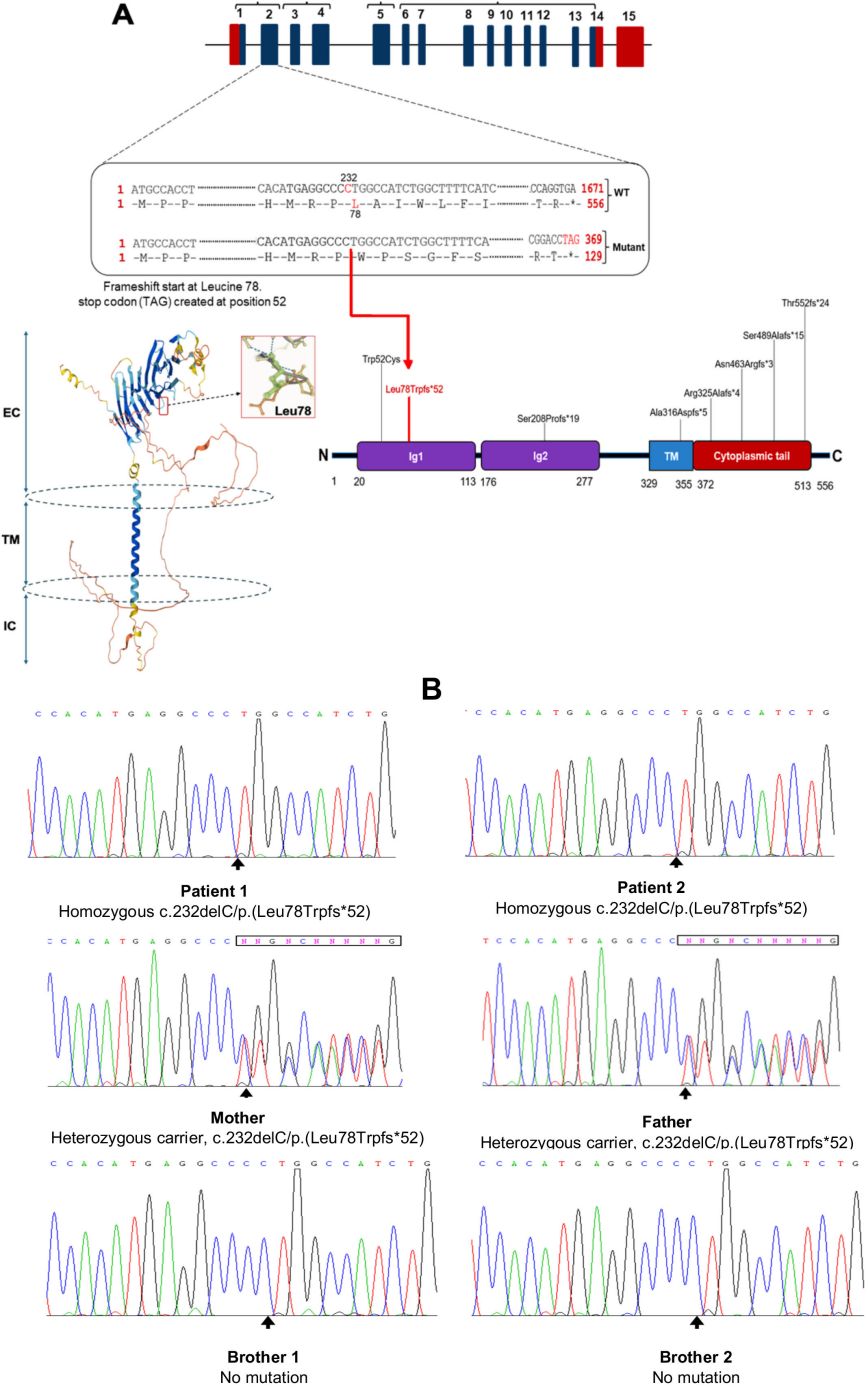
Ahomozygous nucleotide deletion in CD19 gene disrupts the reading frame, resulting in early termination of translation. **(A)** Schematic overview of the CD19 gene, the 3D model of the human CD19 protein obtained from the AlphaFold Protein Structure Database via RCSB PDB (www.rcsb.org/structure/AF_AFP15391F1) (23), and its functional domains with previously reported mutations; EC, extracellular; TM, transmembrane; IC, intracellular. Both patients carried a homozygous cytosine deletion at nucleotide position 232 in exon 2 of the CD19 gene, resulting in a frameshift at leucine 78 and the introduction of a premature stop codon (TAG) 52 amino acids downstream. TM, transmembrane domain; Ig, immunoglobulin-like domain. **(B)**. DNA Sanger sequencing confirmed CD19 nucleotide deletion (c.232delC) in patients and family members.

To identify the underlying molecular defect, whole exome sequencing (WES) was performed for patient 1, the eldest affected sibling. Bioinformatic analysis revealed a homozygous deletion of a cytosine nucleotide in exon 2 of the *CD19* gene at cDNA position 232 (GenBank accession: NM_001770.6), designated as c.232delC. At the genomic level, this corresponds to NC_000016.10:g.28932489del and based on the human genome assembly GRCh37/hg19, the chromosomal position is chr16:28943810delC. This variant was queried in the Genome Aggregation Database (gnomAD) v2.1.1, which is compatible with the hg19 reference genome, and was not reported in either the Exomes or Genomes datasets. The c.232delC mutation is predicted to cause a frameshift resulting in a premature stop codon in exon 2 (p.Leu78Trpfs*52), initiating at codon 78 and leading to a stop codon 52 amino acids downstream (**Figure 3A**). This frameshift is expected to result in nonsense-mediated mRNA decay (NMD) and complete loss of functional CD19 protein expression. According to the American College of Medical Genetics and Genomics (ACMG) criteria, the variant is classified as “pathogenic” (24).

The mutation was confirmed by Sanger sequencing, which verified its segregation with the disease phenotype. Family screening revealed that both parents were heterozygous carriers of the mutation. The symptomatic younger brother (Patient 2) was also found to be homozygous for the same mutation, while the two remaining asymptomatic siblings tested negative for the variant (**Figure 3B**).

### CD19-complex members expression

**Figure 4:**
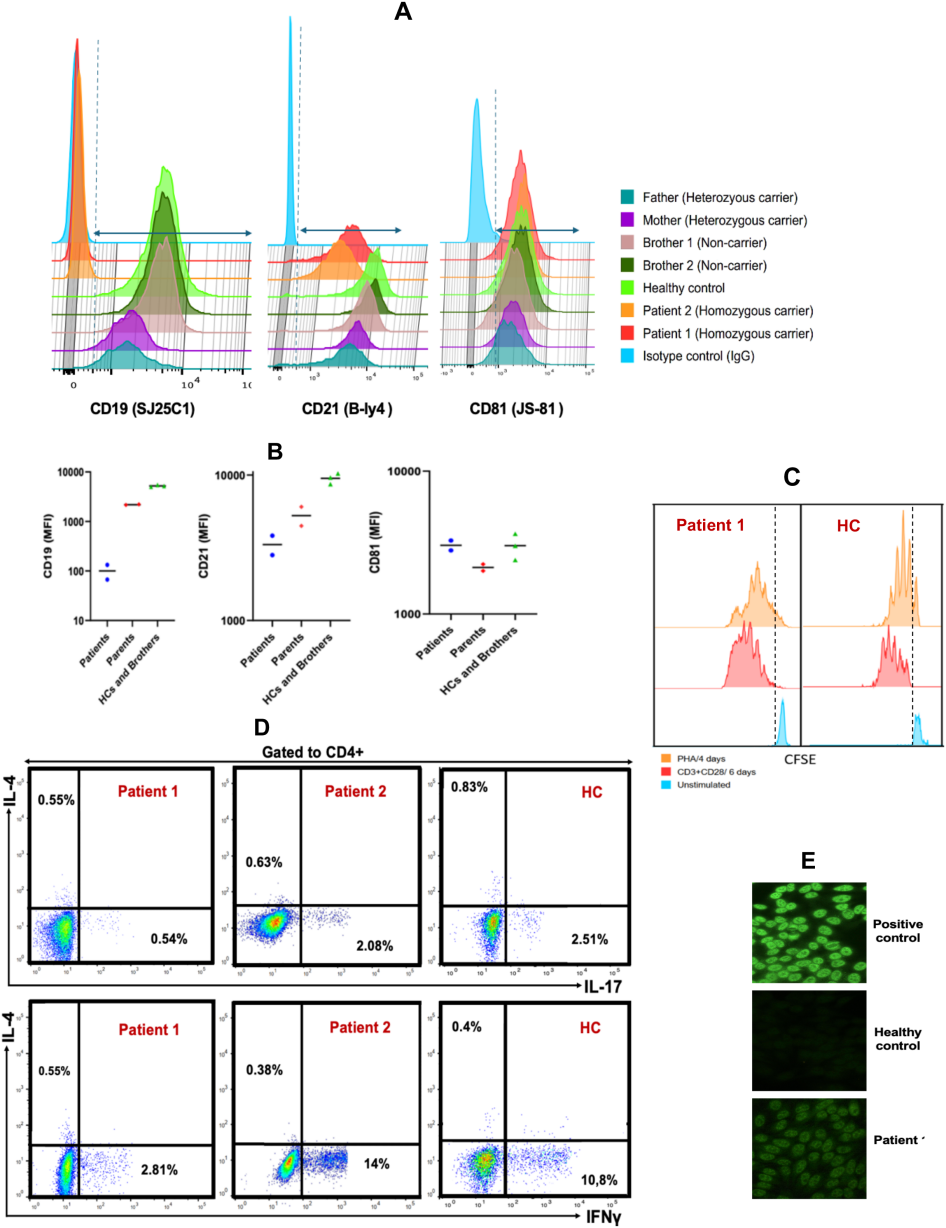
Analysis of B-cell Surface Markers, T-cell Cytokine Production, and Proliferative Responses in CD19-deficient Patients and Relatives **(A)**. Flow cytometric histograms illustrating membrane expression of CD19, CD21, and CD81 on CD20^+^ B cells from patients, healthy controls, siblings, and parents. **(B)**. Comparison of the median fluorescence intensity (MFI) of CD19, CD21, and CD81 expression on CD20^+^ B cells among patients, healthy controls, parents, and siblings. **(C)**. T cell proliferation following 4-day stimulation with phytohemagglutinin A (orange), 6-day stimulation with anti-CD3/CD28 antibodies (red), and unstimulated control (blue). **(D)**. Frequencies of IL-17A-, IFN-γ-, and IL-4–producing CD4^+^ T cells in patient and healthy control samples after 5-hour stimulation with PMA/ionomycin in the presence of monensin. **(E)** Serum from patient 1 exhibited a speckled antinuclear antibody (ANA) pattern by indirect immunofluorescence (IIF) at a titer of 1:160, compared to a positive control with a titer of 1:1000 and a negative result in the healthy control.

The expression of CD19 multimeric complex proteins on B lymphocytes was evaluated by flow cytometry in the affected siblings, their unaffected siblings, parents, and healthy controls (**Figures 4A, B**; **Supplementary Figure 1**). CD19 surface expression was completely absent in both affected siblings, while it was slightly reduced in their heterozygous carrier parents compared to non-carrier siblings and healthy controls. CD21 expression was reduced, but not absent, in both affected patients, whereas CD81 expression remained normal. CD225 expression was not assessed due to lack of validated antibodies.

These findings confirm that the homozygous c.232delC (p.Leu78Trpfs*52) mutation results in a complete loss of CD19 surface expression on B cells in affected individuals, with a partial reduction observed in heterozygous carriers. Moreover, the absence of CD19 appears to impair the expression of CD21, while not affecting CD81, suggesting that CD19 is critical for the stability or surface expression of certain components of the CD19 complex.

### Functional studies

*In vitro* T-lymphocyte proliferation in response to mitogens (PHA) and to anti-CD3/anti-CD28 stimulation was normal (**Figure 4C**). Furthermore, intracellular cytokine staining following CD4+ T cell stimulation showed no abnormalities in the frequencies of CD4+IFNγ+ (Th1), CD4+IL-4+ (Th2), and CD4+IL-17A+ (Th17) cells compared to age- and sex-matched healthy controls (**Figure 4D**). These results indicate that T cell function is preserved, making a primary T cell defect an unlikely cause of the patients’ recurrent infections.

### Autoreactive antibody levels

Autoimmune manifestations occur in approximately 30% of individuals with Common Variable Immunodeficiency (CVID) and represent a significant contributor to morbidity and mortality in this population (25–27). Similarly, the presence of autoantibodies has been documented in patients with CD19 deficiency and has been associated with the development of autoimmune features (9, 14, 28). To explore whether the c.232delC (p.Leu78Trpfs*52) mutation was associated with the production of autoreactive antibodies, both affected siblings were screened for organ-specific and non-organ-specific autoantibodies.

Patient 1 showed a speckled pattern of antinuclear antibodies (ANA) on indirect immunofluorescence (IIF) at a low titer (1:160) (**Figure 4E**), while antibodies against extractable nuclear antigens (ENA) and double-stranded DNA (dsDNA) were negative. Notably, anti-F-actin antibodies were slightly elevated (23 U/mL; reference: <20 U/mL), a finding suggestive of a predisposition to type I autoimmune hepatitis. In contrast, Patient 2 exhibited markedly elevated levels of anti-F-actin (39 U/mL) and anti-soluble liver antigen (SLA) antibodies (60 U/mL; reference: <20 U/mL), both serologic markers associated with autoimmune hepatitis. Despite these findings, liver function tests remained within normal ranges in both patients.

## Discussion

CD19 deficiency is associated with a variable clinical phenotype. While some patients present with symptoms during the first decade of life (9, 14, 15, 17, 29), others are diagnosed later, typically with hypogammaglobulinemia and impaired B cell function (9, 30) (**Table 2**). Recurrent upper and lower respiratory tract infections are the most commonly reported manifestations. However, a broader spectrum of clinical features has been documented, including IgA nephropathy progressing to end-stage renal disease (16), conjunctivitis (31), glomerulonephritis and hematuria (11, 14, 31), IgA vasculitis (11), meningitis (31), and thrombocytopenia (11).

**Table 2:** Baseline Characteristics and Laboratory and Clinical Findings in Patients with CD19 Deficiency, Including Previously Reported Cases.

Patient Characteristic	This paper		Van Zelm (9, 17) et al. and Artac et al. (25)						Kanegane et al. (14)	Vince et al. (16)		Skendros et al. (15)	Walker et al (30)
	P1	P2	P3	P4	P5	P6	P7	P9	P10	P11	P12	P13	P14
cDNA mutation	c.232delC		c.971dup		c.1386_1387del			c.156G > C	c.947-1G>T, aberrant splicing with exon 6 skipped	c.1464delC	c.1653_1671 + 9delins23bp	c.947-1G>T, aberrant splicing with exon 6 skipped	c.622del
Protein change	p.Leu78Trpfs*52		p.Arg325Alafs*4		p.Asn463Argfs*3			p.Trp52Cys	p.Ala316Aspfs*5	p.Ser489Alafs*15	p.Thr552fs*24	p.Ala316Aspfs*5	p.Ser208Profs*19
Sex	Female	Male	Female	Male	Male	Female	Female	Male	Male	Female	Female	Male	Female
Age at onset	8m	5m	1y	6m	7y	6y	5y	6y	5y	Infant	13y	3m	10y
Age at evaluation	10y	5m	10y	12y	35y	33y	49y	6y	8y	11y	31y	11y	30y
Consanguinity	Yes		Yes		No			Yes	No	Unknown	Yes	Unknown	Yes
Ancestry	Algeria		Turkey		Colombia			Morocco	Japan	Kurdish	Morocco	France	United Kingdom
ENT infections	Yes	Yes	Yes	Yes	Yes	Yes	Yes	Yes	Yes	Yes	Yes	Yes	Yes
Pulmonary infections	Yes	Yes	Yes	Yes	Yes	Yes	Yes	Yes	Yes	/	Yes	Yes	Yes
Gastro-intestinal infections	/	/	/	/	Yes	Yes	Yes		Yes	Yes (giardiasis)	/	/	Yes
Mucocutaneous infection	Cutaneous Herpes, Fungal skin infections	/	/	/	Bacterial conjunctivitis,	Herpes zoster infection, conjunctivitis, dacryocystitis	Skin abscesses, conjunctivitis,	Skin infections	/	/	/	/	/
Other infections & manifestations	Urinary tract infections, pyelonephritis	/	Meningitis, Hematuria,	/	/	/	/	/	Pyelonephritis, thrombocytopenia	Meningitis	Hematuria, IgA Nephropathy, end-stage renal disease, failure to thrive	Chronic obstructive pulmonary disease, atopy	/
Autoimmunity & autoantibodies	glomerulo-nephritis, anti-F-actin (+), ANA (+)	Anti-F-actin (+)	glomerulo-nephritis ANA (+) SSA (+)	ANA (–)	(–)	(-)	(-)	(-)	N. A	(-)	ANA (+) but anti-DNA and anti-ENA (-)	ANA (-)	ANA (-)
IgG (mg/L)	436 (↘)	158 (↗)	325 (↘)	(↘)	204 (↘)	198 (↘)	256 (↘)	300 (↘)	249 (↘)	230 (↘)	493 (↘)	440 (↘)	350 (↘)
IgA (mg/L)	144 (N)	86.4 (↗)	292 (↘)	(↘)	18 (↘)	7 (↘)	19 (↘)	50 (N)	10 (↘)	125 (N)	350 (N)	(N)	(N)
IgM (mg/L)	42 (N)	45.4 (N)	25 (↘)	(N)	47 (↘)	30 (↘)	63 (N)	40 (↘)	18 (↘)	35 (↘)	135 (N)	40 (↘)	(N)
Lymphocytes (cell/mm^3^)	5124 (N)	1869 (↘)	4480 (↗)	(N)	2182 (N)	2508 (N)	2059 (N)	1630 (↘)	N.A	2,745 (N)	1,440 (N)	N.A	(N)
CD3+T cells (cell/mm^3^)	4447 (↗)	1482 (↘)	3270(↗)	(N)	1520 (N)	1855 (N)	1384 (N)	1107 (↘)	1775 (N)	2,141 (N)	1,152 (N)	(N)	(N)
CD4+T cells(cell/mm^3^)	2086 (↗)	859 (↘)	1792 (N)	(N)	713 (N)	1070 (N)	620 (N)	637 (↘)	1064 (N)	1,125 (N)	590 (N)	N.A	(N)
CD8+T cells (cell/mm^3^)	1925 (↗)	537 (↘)	1478 (N)	(N)	720 (N)	692 (N)	696 (N)	353 (↘)	781 (N)	796 (N)	533 (N)	N.A	(N)
CD4+/CD8+ Ratio	1.08 (↘)	1.60 (N)	1.21 (↘)	N.A	0.99 (↘)	1.54 (N)	0.89 (↘)	1.80 (N)	1.36 (↘)	1.41 (↘)	1.10 (↘)	N.A	(N)
CD20+ B cells (cell/mm^3^)	562 (N)	266 (N)	806 (N)	(N)	286 (N)	521 (N)	268 (N)	321 (N)	N.A	300 (N)	61 (↘)	11% (N)	(N)
CD56+NK cells (cell/mm^3^)	115 (N)	119 (N)	313 (N)	(N)	277 (N)	348 (N)	288 (N)	156 (N)	N.A	184 (N)	23 (↘)	16% (N)	(N)
mzB cells (%)	2.29 (↘)	1,67 (N)	N.A	(↘)	(↘)	(↘)	(↘)	3 (↘)	5.4 (↘)	8 (N)	22 (↗)	2 (↘)	(↘)
smB cells (%)	2.50 (↘)	0.44 (N)	N.A	(↘)	(↘)	(↘)	(↘)	3 (↘)	7.6 (↘)	2 (↘)	12 (↗)	1 (↘)	(↘)
Transitional B cells (%)	5.64 (N)	55,6 (↗)	N.A	N.A	N.A	N.A	N.A	3 (↘)	N.A	0 (↘)	0 (↘)	5 (N)	N.A
Plasmablast (%)	0.43 (↘)	0,34 (N)	N.A	N.A	N.A	N.A	N.A	N.A	N.A	0 (↘)	0 (↘)	6 (↘)	N.A
CD21^low^CD38^low^B cells (%)	0.21 (N)	0,024 (N)	N.A	N.A	N.A	N.A	N.A	N.A	N.A	0.4 (N)	2.9 (N)	1 (N)	N.A
*In vitro* B response	N.D	N.D	(↘)	N.A	(↘)	(↘)	(↘)	(↘)	(↘)	N.A	N.A	N.A	(↘)
Antibody response to vaccine	(↘)	N.A	(↘)	(↘)	(↘)	(↘)	(↘)	(↘)	(↘)	(↘)	(N)	(↘)	(↘)

y, year; m, month; Ig, immunoglobulin; mzB, marginal zone B cells; smB, switched memory B cells; (-), negative; (↗), increased; (↘), decreased; (N), within normal limits; N.A, not available. N.D, not done; ENT, Ear, Nose and Throat; ANA, anti-nuclear autoantibodies; ENA, extractable nuclear antigen.

We report two Algerian siblings with CD19 deficiency who presented with a history of recurrent infections. The clinical features observed in both patients were largely consistent with those previously described in CD19-deficient individuals (29) with the exception of a few episodes of fungal and viral infections in patient 1, which resolved following appropriate treatment.

Only patient 1 showed a decrease in serum IgG levels along with impaired specific antibody production, consistent with the other CD19-deficient patients. In contrast, her younger brother (patient 2) exhibited significantly elevated serum levels of IgG and IgA rather than hypogammaglobulinemia. However, it is important to note that he was only 3 months old at the time of measurement, and the persistence of maternal IgG up to 6 months of age must be taken into account (32). Beyond the variability in clinical phenotype, CD19 deficiency also shows heterogeneity in age of onset—ranging from infancy to adolescence or early adulthood—as well as in serum immunoglobulin levels (IgG, IgA, IgM), which tend to decline progressively with age (32).

By analyzing the exome data of the index patient within the candidate disease locus, and subsequently validating the candidate variant through Sanger sequencing in affected individuals and their parents, we identified a biallelic nucleotide deletion in exon 2 of the *CD19* gene (c.232delC). This deletion causes a frameshift mutation (p.Leu78Trpfs*52), which could trigger nonsense-mediated mRNA decay (NMD). It is important to note that RNA-based analysis could not be performed due to limited resources. This constitutes a significant limitation of our study, particularly for evaluating this mechanism. We suggest that RNA-based functional analyses be considered in the future to validate this mechanism. Nevertheless, NMD is known to selectively eliminate mRNA transcripts containing premature stop codons, such as those resulting from nonsense or frameshift mutations (33, 34).

Functional analysis revealed a complete absence of CD19 surface expression on B cells from the affected siblings, while their parents showed reduced levels. It has been reported that two of the three intracytoplasmic C-terminal tyrosine residues of CD19 are essential for signal transduction (1, 35). Moreover, CD19 forms a signaling complex with complement receptor 2 (CD21), the tetraspanin CD81, and CD225 (Leu-13), which is crucial for transmitting signals from C3d-tagged antigens to the B cell receptor (BCR) and CD21 (1, 2, 8, 35). In our patients, peripheral B cells displayed normal expression levels of CD81, but CD21 expression was reduced—a finding consistent with previous reports in other CD19-deficient individuals (9, 14, 16, 17, 30). These observations support the notion that CD19 is essential for the stable expression of CD21, but not CD81. Conversely, CD19 surface expression is highly dependent on CD81. This interdependence was demonstrated in a patient harboring a homozygous G>C substitution in exon 6 of the *CD81* gene (c.561 + 1G>A), which resulted in a frameshift and premature stop codon (p.Glu188Metfs*13). In this case, both CD19 and CD81 were absent on the patient’s B cells, with reduced expression observed in heterozygous carriers (11).

CD81—the first tetraspanin identified—has been proposed to play two key roles. First, it binds CD19 via its ectodomain and facilitates its trafficking to the cell surface through the secretory pathway (36, 37). Second, CD81 regulates B cell signaling by controlling the localization of CD19 on the plasma membrane during B cell activation (38).

B cell receptor (BCR) signaling is finely tuned by surface coreceptors such as CD19 and CD22 (8). Upon antigen binding, the BCR initiates a signaling cascade through the phosphorylation of immunoreceptor tyrosine-based activation motifs (ITAMs) located in the cytoplasmic tails of Ig-α (CD79A) and Ig-β (CD79B), mediated by Src family tyrosine kinases including LYN, SYK, FYN, and BLK (39–44). Activated SYK and other downstream kinases further phosphorylate key adaptor and signaling molecules such as BLNK, BCAP, and CD19 (45). Once phosphorylated, CD19 acts as a scaffold to recruit various effectors—including phosphoinositide 3-kinase (PI3K), phospholipase C gamma (PLCγ), NCK, BAM32, Bruton’s tyrosine kinase (BTK), VAV1, and SHC—to the plasma membrane. These interactions promote the formation of large multiprotein complexes that ultimately trigger a robust influx of extracellular calcium (Ca²^+^), which is essential for full B cell activation (45–48) (**Figure 1**).

In CD19-deficient (CD19^-^/^-^) B cells, calcium flux is significantly reduced, as demonstrated in several studies (9, 17, 18, 49). This finding supports the conclusion that the impaired B cell responses observed in CD19-deficient patients result from compromised signaling capacity, ultimately leading to both quantitative and qualitative deficiencies in immunoglobulin production.

Interestingly, elevated levels of autoantibodies targeting nuclear and liver autoantigens—including antinuclear antibodies (ANA), anti-F-actin, and anti–soluble liver antigen (anti-SLA) antibodies—were detected in our patients. Similar findings have been reported in other individuals with CD19 deficiency (9, 14, 16, 50), suggesting that autoimmunity is a common feature of this condition. The underlying mechanisms driving the high prevalence of autoantibodies in CD19 deficiency remain unclear. It is postulated that, during B cell differentiation and maturation—particularly prior to the onset of somatic hypermutation—mechanisms exist to eliminate or inactivate autoreactive B cells and thereby prevent autoantibody production (51, 52). In CD19-deficient patients, this central tolerance checkpoint may be impaired, allowing autoreactive clones to escape deletion and produce a wide range of autoantibodies against self-antigens.

Moreover, CD19 has been proposed to play a key role in BCR-mediated signaling in B cell precursors. By enhancing the strength of the BCR signal, CD19 contributes to the negative selection of autoreactive B cells, helping to maintain immune tolerance  (5). Further studies are warranted to clarify the precise role of CD19 in the development of autoimmune manifestations and to explore its potential involvement in broader autoimmune disorders.

Strikingly, the incidence of nephropathy among CD19-deficient patients appears to be relatively high. This complication was observed in our patient 1 and has also been reported in 3 out of 11 other documented cases. Supporting this clinical observation, Watanabe et al. (16) demonstrated a similar phenomenon in a CD19-deficient murine lupus model (CD19^-^/^-^ NZB/W mice), generated from a cross between New Zealand Black and New Zealand White F1 hybrids. In this model, pathological features of nephritis—including glomerulonephritis and interstitial nephritis—developed significantly earlier, and overall survival was markedly reduced compared to wild-type controls. The study further revealed that CD19^-^/^-^ lupus-prone mice failed to produce a subset of interleukin-10 (IL-10)–producing regulatory B cells (Bregs), which are known to play a critical role in immune regulation. The absence of these Bregs likely contributes to the early onset and severity of nephritis observed in CD19-deficient mice and may similarly help explain the increased prevalence of renal involvement in CD19-deficient patients.

The eldest patient 1 experienced a viral skin infection of herpetic origin, along with several episodes of mucocutaneous fungal infections. These fungal infections responded well to topical treatment and did not recur after discontinuation of therapy. The occurrence of these opportunistic infections may be partially attributed to the family’s socio-economic conditions, which could contribute to increased susceptibility. Furthermore, the patient suffered from malnutrition, resulting in significant weight loss (–3 SD), a factor that can compromise immune defenses against opportunistic pathogens. Notably, the frequency of interleukin-17–producing CD4^+^ T cells (Th17 cells) which play a key role in antifungal immunity, was within normal limits in this patient, suggesting that her susceptibility to fungal infections was likely not due to Th17 cell deficiency.

## Conclusion

In this report, we describe a case study of two siblings with CD19 deficiency caused by a novel homozygous mutation in the *CD19* gene, resulting in nonsense-mediated mRNA decay. The clinical and immunological features observed in both patients were consistent with those previously reported in the literature. Defective CD19-mediated signaling impaired normal B cell responses to antigenic stimulation, leading to poor antigen-specific antibody production. Notably, no additional complications commonly associated with common variable immunodeficiency (CVID) such as granulomatous disease or lymphoproliferation were observed in either patient. These findings support the classification of CD19 deficiency as a distinct and well-defined subset within the spectrum of predominantly antibody deficiencies.

## Data Availability

The raw data supporting the conclusions of this article will be made available by the authors, without undue reservation.
