# Supplementary material for: A novel homozygous frameshift mutation likely causing nonsense-mediated mRNA decay in an Algerian kindred with CD19 complex deficiency
Source: Front Immunol. 2025 Sep 5;16:1634146. doi: 10.3389/fimmu.2025.1634146 (PMC12446367; doi:10.3389/fimmu.2025.1634146)
Supplement: Supplementary file 1 [file Table1.docx]

**Supplementary table S1:** Flow cytometry panels used in lymphocyte immunophenotyping

| Panel | Target proteins |
| --- | --- |
| T, B and NK cell panel | CD3-FITC, CD4-PE-Cy7, CD8-APC-Cy7, CD16-PE, CD19-APC, CD45-PerCP-Cy5.5, CD56-PE |
| Extended T cell panel | CD4- PE-Cy7, CD8-PerCP, CD31-APC-Cy7, CD45RA-FITC, CD45RO-PE, CCR7-BV-510 |
| Extended B cell panel | CD20-V450, CD21-FITC, CD24-PE-Cy7, CD27-PE, CD38-APC-C7, IgD-PerCP-Cy5.5 |
| Regulatory T cell panel | CD3-PerCP-Cy5.5, CD4-PE-Cy7, CD25-APC, CD127-PE, FoxP3-Alexa fluor^®^ 488 |
| T follicular helper cells | CD3-PerCP-Cy5.5, CD4-APC, CD45RA-FITC, CD45RO-PE-Cy7, TCRαβ-FITC, hCXCR5-PE |
| Lymphocyte proliferation assay | CFSE, 7AAD, CD4-APC |
| CD19, CD21 and CD81 expression | CD19-FITC, CD20-V450, CD21-PE, CD81-APC-Cy7 |

**APC**: allophycocyanine; **BV**: brilliant violet; **Cy**: cyanine; **CCR**: C-C chemokine receptor type 7**; CD**: cluster of differentiation; **CFSE**: Carboxyfluorescein succinimidyl ester; **FITC**: Fluorescein isothiocyanate; **PE**: phycoerythrin; **PerCp**: peridinin-Chlorophyll-protein; **7AAD**: 7-aminoactinomycin D
