# Supplementary material for: A novel homozygous frameshift mutation likely causing nonsense-mediated mRNA decay in an Algerian kindred with CD19 complex deficiency
Source: Front Immunol. 2025 Sep 5;16:1634146. doi: 10.3389/fimmu.2025.1634146 (PMC12446367; doi:10.3389/fimmu.2025.1634146)
Supplement: Supplementary file 2 [file Table2.docx]

**
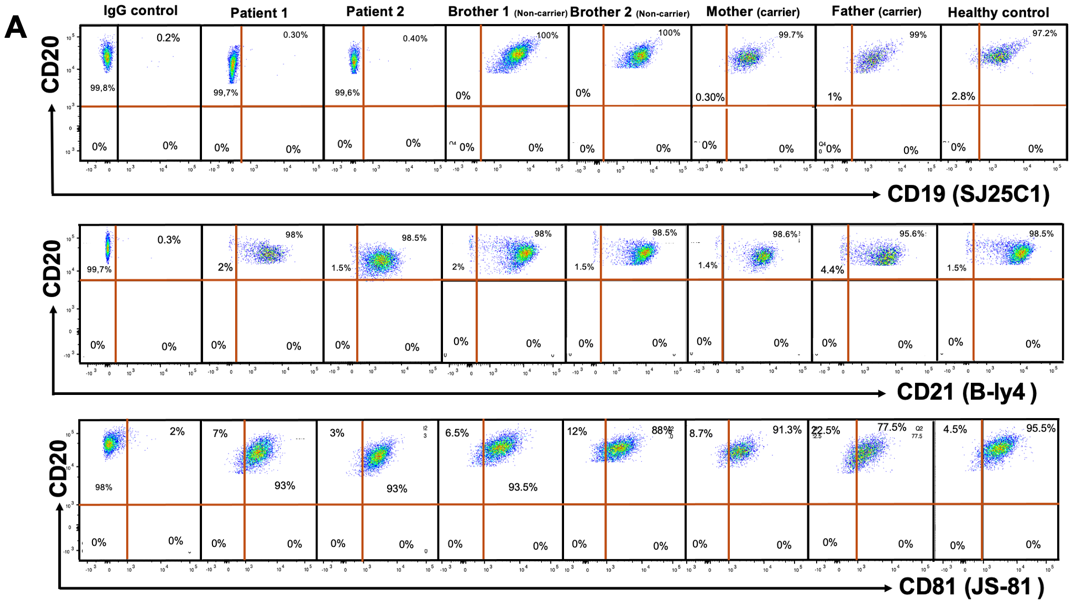
Supplementary figure 1:** Flow cytometric dot plots illustrating membrane expression of CD19, CD21, and CD81 on CD20⁺ B cells from patients, healthy controls, siblings, and parents.
